# Encapsulated biocides in facade materials impact leaching and UV stability, resulting in lower aquatic toxicity of the eluates

**DOI:** 10.1007/s11356-025-36647-2

**Published:** 2025-06-25

**Authors:** Moritz Nichterlein, Nadine Kiefer, Jenny Hohner, Dominik Stapf, Madeleine Schatz, Matthias Noll, Stefan Kalkhof

**Affiliations:** 1 Institute for Bioanalysis, University of Applied Sciences Coburg, Coburg, Germany; 2 Institute for Analytical Chemistry, Universität Leipzig, Leipzig, Germany; 3 SGS Analytics Germany GmbH, Augsburg, Germany; 4 Limbach Analytics GmbH, Leipzig, Germany; 5 Proteomics Unit, Fraunhofer Institute for Cell Therapy and Immunology, Leipzig, Germany

**Keywords:** Film preservative, In-can preservative, Immersion test, Irradiation, Facade, Ecotoxicity, Luminescent bacteria, Green algae

## Abstract

In the construction or reconstruction of building facades, user-friendly, dispersion-based renders and paints, which contain organic polymers, are increasingly being used. These materials are susceptible to microbial colonization, potentially causing both esthetic and structural damages. To prevent this, biocides are often added, which in turn can have harmful environmental effects after leaching induced by wind-driven rain. To reduce leaching rates, biocides are widely utilized in encapsulated form. In this study, we investigated, using test facades and standardized leaching with and without sunlight irradiation, whether and to what extent sunlight-caused UV exposure additionally impacts the leaching of encapsulated biocides compared to unencapsulated biocides, and how this affects the chemical composition as well as the toxicity of the resulting eluates. Our results show that encapsulation significantly improves the UV stability of the biocides 2-Octyl-1,2-thiazol-3-one (OIT) and 4,5-Dichloro-2-octyl-1,2-thiazol-3-one (DCOIT), thereby extending their effectiveness in facade materials, while Terbutryn is only slightly affected. Furthermore, encapsulation leads to a reduction in the concentrations of the leached biocides, both without and especially with irradiation, along with a corresponding decrease in the toxicity of the eluates towards aquatic model organisms, luminescent bacteria, and green algae. These findings emphasize the relevance of encapsulation as an effective strategy for improving the sustainability and safety of biocides in facade materials, as well as the need to consider the influence of UV irradiation in toxicological evaluations of renders and paints.

**Supplementary Information:**

The online version contains supplementary material available at 10.1007/s11356-025-36647-2.

## Introduction

Facades are permanently exposed to the environment and consequently vulnerable to colonization by microorganisms such as bacteria, fungi, and algae. As microbial colonization progresses, it leads not only to esthetic impairments caused by the pigmentation of biofilms and individual organisms but, in extreme cases, can also harm and impact the structural integrity of the building (Gaylarde et al. 2011; Malathi and Devanathan 2017). In recent years, dispersion-based ready-to-use renders and paints have become predominant (Burkhardt et al. 2011; Paijens et al. 2020) due to their user-friendly and practical application. However, these highly engineered and complex materials commonly contain organic polymers, such as binders, fillers, or plasticizers, which can serve as nutrient sources for microorganisms (Gaylarde et al. 2011; Malathi and Devanathan 2017). Consequently, such facade materials would only provide inadequate intrinsic protection against microbial infestation without the implementation of additional microbial inhibiting techniques. As a result, various product technologies including the application of hydrophobic (Prudente et al. 2024) or photoactive (Wei et al. 2023; Prudente et al. 2024) coatings or the addition of film preservatives (Reiß et al. 2021; Schoknecht et al. 2009), have been developed and commercialized by paint and render manufacturers to prevent a broad diversity of microbial colonization and delay those microbial community members that still are capable to colonize.

Coating systems or additives incorporated into the construction matrix can be applied to create facade surfaces with hydrophobic properties to reduce moisture and microbial adhesion. Additionally, the water-repellent lotus effect removes attached particles and potential substrates from the surface while also reducing moisture within the facade matrix, an essential factor for microbial proliferation. However, this technology is not yet considered sufficiently durable and instead requires extensive maintenance, as mechanical (scratches, abrasion, vibrations) and environmental (UV radiation, precipitation, frost, contamination) factors can alter the surface properties, affecting the required parameters and compromising its effectiveness in preventing microbial growth (Yuranova et al. 2007; Prudente et al. 2024; Mahltig et al. 2008).

Another strategy for preventing microbial colonization of facades is the incorporation of additive photoactive substances in the outer house wall matrix. Typically, semiconductors with a crystalline structure are employed as photocatalysts in construction materials, leading to the formation of hydroxyl radicals (HO^•^) and hydroperoxyl radicals (HO_2_^•^) upon sunlight irradiation (Wei et al. 2023). These highly reactive radicals decompose adhering dirt particles and have an antimicrobial activity. Photoactive TiO_2_ and ZnO are probably the most studied and applied photocatalysts in building materials (Prudente et al. 2024). TiO_2_ is chemically stable, cost-effective, and non-toxic and, as a white pigment, contributes significantly to the coloring of house facades, making it indispensable in many paints and renders (Allen et al. 2008). ZnO shows similar advantages and disadvantages as TiO_2_, with the additional benefit of antibacterial properties independent of light exposure. ZnO, also a white pigment, acts as a hydration retarder and improves the mechanical stability of building materials (Prudente et al. 2024). Since both TiO_2_ and ZnO can only achieve their maximum photoactive and, therefore, self-cleaning potential in the UV range, their use may not be efficient all year round and may require adaptation or combination with other technologies.

The use of biocides is a well-established and market-applied approach for the microbial protection of dispersion-based ready-to-use renders and paints. The active substances are added to the product matrix as additives with up to 0.5% (w/w) of the total product (Jämsä et al. 2013; Burkhardt et al. 2011; Nordstierna et al. 2010) during the manufacturing process so that the building materials are protected against microbial infestation as wet formulation during storage and later, after application, as part of the facade (Kiefer et al. 2024; Reiß et al. 2021). A combination of differently acting bactericides, fungicides, and algaecides is typically utilized to achieve comprehensive microbial protection (Burkhardt et al. 2011). Biocides intended to maintain the storage stability of building materials are classified as in-can preservatives, whereas biocides for the protection of the facade matrix are classified as film preservatives. In-can preservatives, designed to protect the wet product formulation from microbial growth during the storage period, typically have a lower molecular mass, are more water-soluble, and have a relatively short half-life (Kiefer et al. 2024). In contrast, film preservatives exhibit a higher molecular weight, a lower water solubility, and a longer lifespan to prevent or at least delay microbial colonization of the facade for extended periods (Kiefer et al. 2024). Biocides require a certain level of water solubility and mobility within the facade matrix to effectively combat living microorganisms. As a result, biocides are leached out of the facade by wind-driven rain and enter the environment, where they can have unspecific effects on terrestrial and aquatic organisms. Biocides have been detected in various overflow basins, water treatment plants, rivers, and soil compartments (Paijens et al. 2020; Hensen et al. 2018; Bollmann et al. 2017a). The toxicological impact of biocide-containing facade runoff on soil and water organisms has also been demonstrated in several studies (Kiefer et al. 2024; Vermeirssen et al. 2018; Reiß et al. 2025). Increasing the biocide concentration can enhance the duration and efficacy against microbial proliferation (Nordstierna et al. 2010). However, higher biocide quantities result in extended toxicological pressure on the environment. Consequently, the application of biocides in construction materials within the European Economic Area is regulated and limited by the European Biocide Products Regulation (European Parliament 2012).

To prolong the availability of an effective antimicrobial concentration of biocides on facade surfaces while simultaneously reducing the required initial concentration, film preservatives are commonly used in encapsulated form nowadays (Vermeirssen et al. 2018). Encapsulation of active ingredients is a well-established method employed across diverse applications, including the food, pharmaceutical, and agricultural sectors, to protect them from environmental influences and regulate their release behavior (Issayeva et al. 2024; Frydenberg et al. 2022; Szendy et al. 2019). Various encapsulation technologies, such as coacervation, sol–gel, or self-assembly processes, can be utilized depending on the active ingredient and material properties (Arzani and Dos Santos 2022). The selection of an appropriate encapsulation technology depends on various factors, including the physicochemical properties of the active ingredient, the material composition, and the desired product characteristics, requiring a tailored and customized approach (Bergek et al. 2014; Arzani and Dos Santos 2022; Nordstierna et al. 2010). The biocides must be able to tolerate the exposed chemical (solvents, extreme pH values, etc.) and physical (temperatures, gravitational forces, etc.) stresses during the encapsulation process; furthermore, the encapsulated active ingredients should not negatively affect the optical and technical characteristics of the final product (Arzani and Dos Santos 2022). In addition to shielding the biocides from environmental factors, the polymer material must also ensure the adequate release of the active ingredient, typically through passive diffusion, to maintain minimally effective levels against microorganisms (Sørensen et al. 2010; Nordstierna et al. 2010). Despite all challenges, the encapsulation of biocides in building materials offers indispensable advantages. Previous studies have demonstrated that encapsulated biocides are washed out of building material matrices in a reduced and delayed manner (Sørensen et al. 2010; Nordstierna et al. 2010), and the ecotoxicity of biocide-containing facade eluates and leachates on model organisms is reduced by encapsulation (Vermeirssen et al. 2018). Some investigations have also implied that encapsulation may protect against irradiation of the active substances (Sørensen et al. 2010; Edge et al. 2001); however, comprehensive comparative data on the degradation of encapsulated versus non-encapsulated biocides within a facade matrix under UV exposure is still lacking.

Consequently, our study systematically addresses three major research questions:

I. Do encapsulated and non-encapsulated biocides exhibit differential degradation under sunlight irradiation?
II. To what extent does encapsulation reduce biocide leaching and, consequently, decrease toxicity of the eluates?
III. Does additional irradiation of facades containing encapsulated biocides additionally affect the biocide leaching behavior and the resulting toxicity of facade eluates?

Thus, in this study, the effects of sunlight radiation and/or leaching on non-encapsulated and encapsulated biocides incorporated into facade test samples were investigated under laboratory conditions. Additionally, the study evaluated the toxicity of facade eluates from irradiated and non-irradiated facade samples containing either non-encapsulated or encapsulated biocides using aquatic model organisms.

## Materials and methods

### Materials

Different grades of methanol (HPLC gradient grad, LC–MS grade, Synthesis grade, all from Carl Roth, Karlsruhe, Germany) and formic acid (LC–MS grade, VWR International, Leuven Belgium; Synthesis grade, Carl Roth, Karlsruhe, Germany) were applied as LC-eluents and for sample preparation. In addition, ultra-pure water was used, prepared by a Milli-Q Direct-Q 3 UV system (Merck, Darmstadt, Germany).

For biocide quantification, the following analytical standards were purchased: 2-Methyl-1,2-thiazol-3-one:5-Chloro-2-methyl-1,2-thiazol-3-one-mixture (MIT:CMIT, Sigma-Aldrich, Burlington, MA, USA), 1,2-Benzothiazol-3-one (BIT), Terbutryn (TB), 2-Octyl-1,2-thiazol-3-one (OIT), 4,5-Dichloro-2-octyl-1,2-thiazol-3-one (DCOIT), Mecoprop. All mentioned standards, except MIT:CMIT, were obtained from Dr. Ehrenstorfer (Augsburg, Germany). The following stable isotope-labeled (SIL) standards were used for LC–MS analysis: MIT-D3, CMIT-D3, TB-D5 (all from Dr. Ehrenstorfer, Augsburg, Germany), ^13^C_6_-BIT and OIT-D17 (both from Toronto Research Chemicals, Toronto, Canada).

ACTICDE products from Thor (Speyer, Germany) were utilized for the production of biocide-containing facade test samples (render paint system, RPS). The biocides used were individually available, except for the MIT:CMIT-mixture, in encapsulated (cap) and/or non-encapsulated (ncap) form, as dispersion or solution. In-can preservatives MIT, CMIT (ACTICIDE MV), and BIT (ACTICDE BCL 2) were available exclusively in non-encapsulated form, whereas film preservatives TB (ACTICIDE SR 2044 (ncap); ACTICIDE ATA (cap)), OIT (ACTICIDE OTW (ncap); ACTICIDE OTA 20 (cap)), and DCOIT (ACTICIDE DTC (ncap); ACTICIDE DCA 10 (cap)) were available in both non-encapsulated and encapsulated (AMME™ technology) forms.

### Preparation of render and paint formulations and test facade

The paint and render formulations for the production of the test facades were prepared as described by Schoknecht et al. (2013) and Kiefer et al. (2024), whereas the solvent content was replaced by water, and the biocidal products were given according to the desired starting concentration (Supplementary table S1). ACTICDE products were added during the manufacturing process to produce biocide-containing test samples with target concentrations of 15 mg/kg wet weight (ww) MIT:CMIT (1:3), 1000 mg/kg ww BIT, and 500 mg/kg ww of each OIT, TB, and DCOIT in paints and 15 mg/kg ww MIT:CMIT (1:3), 500 mg/kg ww BIT, and 350 mg/kg ww of each OIT, TB, and DCOIT in renders, regardless of whether non-encapsulated or encapsulated film preservatives were applied. In total, three different variants of test facades were prepared and examined, including a control variant without the active addition of biocides (RPS_contr), a variant with in-can preservatives and only non-encapsulated film preservatives (RPS_ncap), and a variant with in-can preservatives and only encapsulated film preservatives (RPS_cap). The wet paint and render formulations as well as the solid facade test samples were produced according to industrial standards at the Dr. Robert Murjahn Institute (Ober-Ramstadt, Germany).

### Study design

**Fig. 1:**
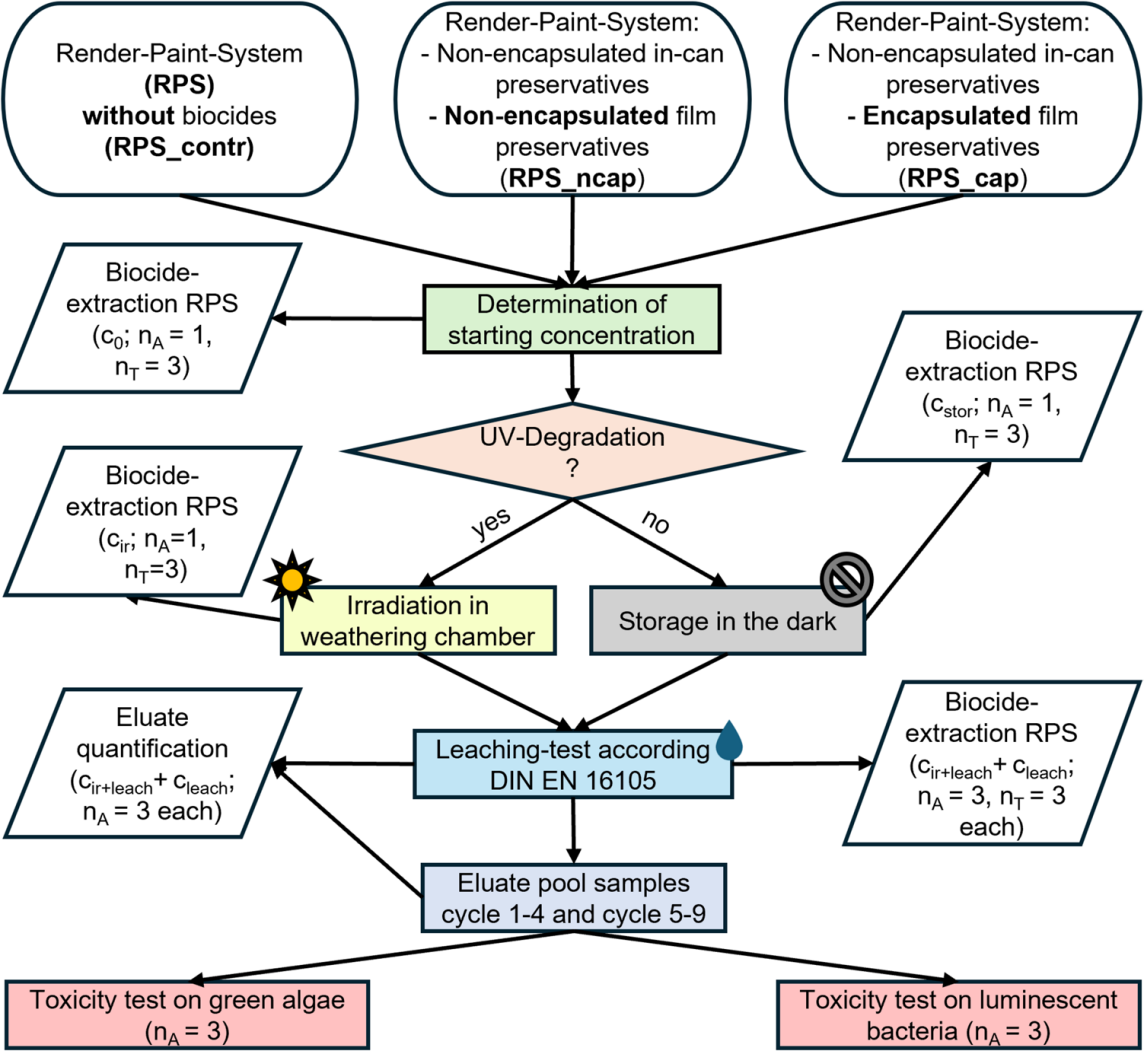
Overview of the sample treatments and analysis. After determining the initial biocide concentration (c_0_) in the facade test samples (RPS), the samples were first stored (stor) or irradiated (ir). Afterwards, the DIN EN 16105 immersion test was performed (leach). The combined eluates from the immersion cycles 1–4 and 5–9 were tested to determine the toxicity to green algae (DIN 38412 L33:1991–03) and luminescent bacteria (DIN EN ISO 11348–2). Samples were analyzed as analytical (n_A_) and/or technical replicates (n_T_)

Using various facade test samples containing non-encapsulated in-can preservatives and either encapsulated or non-encapsulated film preservatives, the UV degradation, the leaching behavior, and the ecotoxicological effects of generated eluates were investigated and compared on a laboratory scale. To determine the effects of encapsulation on the environmental factors mentioned, the following treatments and analyses were carried out (Fig. 1):

- Determination of the initial concentrations in the facade test samples
  - Biocide extraction from facade test samples without treatment or storage (c_0_)
- Irradiation of facade test samples in the weathering chamber
  - Biocide extraction from facade test samples to determine the remaining biocide concentrations after irradiation (c_ir_)
- Storage of facade test samples without irradiation or leaching
  - Biocide extraction from facade test samples to determine the remaining biocide concentrations after storage (c_stor_)
- Leaching of facade test samples without irradiation
  - Biocide extraction from facade test samples to determine the remaining biocide concentrations after leaching (c_leach_) + biocide quantification of the eluates
  - Toxicological test of the eluates (pool samples cycle 1–4 and cycle 5–9) on green algae and luminescent bacteria
- Irradiation and leaching of facade test samples
  - Biocide extraction from facade test samples to determine the remaining biocide concentration after irradiation and leaching (c_ir+leach_) + biocide quantification in the eluates
  - Toxicological test of the eluates (pool samples cycle 1–4 and cycle 5–9) on green algae and luminescent bacteria

### Irradiation

The irradiation of the facade specimens was carried out in a weathering chamber (Q-Sun Xe-1, Q-Lab, Saarbrücken, Germany) equipped with a xenon arc lamp and a daylight filter, simulating the sunlight spectrum starting at 295 nm. Samples were placed in the weathering chamber for 14 consecutive days, alternating 6.2 h of irradiation and 17.8 h of incubation in darkness. The total UV (TUV) radiation in the range of 300 to 400 nm was set to 39 W/m^2^ at a maintained temperature of the black panel sensor of 45 ± 2 °C. For passive humidity regulation, water reservoirs were located inside the chamber as well as two sensors for continuous monitoring of air temperature and relative humidity. Non-irradiated reference facades were stored in darkness at room temperature (25.0–26.6 °C; median = 25.8 °C) and room humidity (34–53%; median = 46%) for the same duration.

The radiation treatment was intended to simulate the sunlight influence of two summer weeks on a facade in real time, without precipitation events. The main influencing factors for UV degradation, duration, and irradiation intensity were derived from natural and local weather data to apply realistic testing parameters. Therefore, the average sunshine duration and irradiance from May 2021 to September 2021 were defined as testing parameters for the sample treatment using the weathering chamber. According to weather data from the Deutsche Wetterdienst (German federal authority for weather and climate), the average duration of sunlight from May 2021 to September 2021 was 6.2 h at the closest weather station to Coburg University (weather station ID, 867; location, 50° 18′ 23.8″ N, 10° 58′ 04.4″ E; 4.7 km linear distance to Coburg University of Applied Sciences). The applied irradiation intensity was calculated from recorded data of a photovoltaic system at the Coburg University of Applied Sciences. Between May 2021 and September 2021, the average solar irradiance of the total sunlight spectrum was 4816 Wh/m^2^ per day. Regarding the TUV radiation from 300 to 400 nm, the TUV share was supposed to be about 5% of the total irradiance, verified mathematically based on the integrated area of the sunlight spectrum, at a total irradiance of 1000 W/m^2^. With this assumption, the average TUV radiation (300–400 nm) from May 2021 to September 2021 on 1 day was 240.8 Wh/m^2^, which results in an irradiance of 39 W/m^2^ at a sunlight duration of 6.2 h.

### Leaching test and preparation of eluates for chemical and toxicological analysis

Leaching tests were conducted following the European guideline DIN EN 16105, as previously described by Kiefer et al. (2024), to determine the emissions of encapsulated and non-encapsulated biocides from facade test samples after and without irradiation. For this purpose, the render/paint coating of one sample was exposed to 250 mL deionized water resulting in a contact area of 0.01 m^2^ between the coating and the water surface. A complete leaching test consisted of a total of nine immersion cycles that were carried out on nine consecutive days. One cycle includes 1 h of water exposure, 4 h of drying, and another 1 h of water exposure in a refilled container, performed at a room and water temperature of 20 ± 5 °C. After one complete immersion cycle, the two eluates per day were combined and analyzed. Between and after the completion of the leaching test, the facade test samples were stored at 20 °C with a humidity of 50%. For further tests and analyses, eluates were stored in a refrigerator at 4 °C for a maximum of 7 days until further use. Prior chemical analysis, aliquots of eluate samples were filtered through a polyamide filter with a pore size of 0.25 µm (Carl Roth, Karlsruhe, Germany). In addition, each vial was spiked with 0.1 mg/L (0.01 mg/L for TB-D5) of each available SIL standard.

### Biocide extraction from test facades

Two different extraction methods were applied for sample preparation from facade test samples, one for quantifying in-can preservatives and another for analyzing film preservatives. The sampling and sample preparation protocols for quantifying in-can preservatives by a methanolic extraction were carried out with minor deviations described by Kiefer et al. (2024). Sample preparation for the quantification of film preservatives was conducted using an acidic extraction.

Sampling was performed using a multifunctional tool (Dremel 4250, Bosch Power Tools B.V., Breda, Netherlands) equipped with a diamond-studded cutting edge. Of each test facade, three areas of 2.0 cm × 6.5 cm per position were cut out (Supplementary figure S1). The render/paint samples were mechanically cleaned from attached polystyrene. Afterwards, the extracted material from one test facade was combined into one pooled sample and crushed and sieved to a particle size of ≤ 0.2 mm. The sample weights for the methanolic and acidic extraction were 0.5 g in each case.

For the analysis of in-can preservatives, 20 µg absolute of both deuterated MIT (MIT-D3) and CMIT (CMIT-D3) was added as internal standards to the powdery facade matrix. Afterwards, three ultrasonic extractions (Cycle 1 and 2, 10 mL methanol; Cycle 3, 10 mL methanol:0.1% formic acid 1:1 (v/v), 15 min per cycle; Device: DK 102 P, Bandelin, Berlin, Germany) were performed including centrifugation (5000 rpm, at 4 °C for 15 min; Heraeus Multifuge X1R, Thermo Fisher, Osterode am Harz, Germany) and collection of the supernatants. The combined extracts were concentrated using a rotary evaporator (RV 10 digital, VWR, Germany, and Rotavapor R-100, Büchi, Flawil, Switzerland); furthermore, the solvent was exchanged from methanol to water. Finally, the samples were purified by a reversed-phase extraction (Chromabond C18 Hydra, 6 mL, 500 mg; Macherey–Nagel, Düren, Germany). After sample processing, 0.1 mg/L of BIT-^13^C_6_ was added to the vials of the ready-to-measure solution, composed of 70% ultra-pure water and 30% methanol (v/v).

To quantify the film preservatives, an extraction method using acidic pH was applied. First, samples were weighed into 25 mL volumetric flasks and 3 mL of formic acid (> 99%, synthesis grade) was added. After thorough mixing and an exposure time of about 5 min, 17 mL methanol was added, followed by an exposure time of at least 12 h. Then, samples were incubated in an ultrasonic bath (DK 102 P, Bandelin, Berlin, Germany) for 30 min and the flasks were filled up to a final volume of 25 mL. After a quantitative transfer, the samples were centrifuged (Heraeus Multifuge X1R, Thermo Fisher, Osterode am Harz, Germany) for 15 min and the collected supernatant was filtered through a regenerated cellulose syringe filter with a pore size of 0.2 µm (Carl Roth, Karlsruhe, Germany). As internal standard, 0.5 mg/L of Mecoprop was added to each ready-to-measure solution, consisting of 50% methanol and 50% ultrapure water (v/v).

For each analytical replicate, three technical replicates were prepared. Furthermore, each processed sample series included a biocide-free lime-cement render as blank.

### Instrumental analysis of eluates and extracts

Methanolic facade extracts containing in-can preservatives and all eluate samples were analyzed by LC–MS/MS according to Kiefer et al. (2024). Briefly, measurements were performed on an HPLC-System (1290 Infinity II, Agilent Technologies, Waldbronn, Germany), coupled to a quadrupole time of flight mass spectrometer (QToF 6545, Agilent Technologies, Waldbronn, Germany) with an electrospray ionization (ESI) source (Dual Agilent Jet Stream Electro Spray Ionization (Dual AJS ESI); Agilent Technologies, Waldbronn, Germany) in LC-MS^1^ positive ionization mode. The instrumental parameters for the ion source and the mass spectrometer were set as follows: gas temperature, 325 °C; drying gas, 10 L/min; nebulizer, 20 psig; sheath gas temperature, 400 °C; sheath gas flow, 12 L/min; capillary voltage, 4000 V; nozzle voltage, 200 V; fragmentor, 0–10 min and 20–30 min, 180 V, 10–20 min, 55 V; skimmer, 45 V.

For chromatographic separation, a revered phase C-18 column (Zorbax RRHD Eclipse Plus C18 column, 95 Å, 2.1 mm × 150 mm, 1.8 μm + guard column, 2.1 mm × 5 mm, 1.8 μm; both Agilent, Santa Clara, CA, USA) was utilized at a temperature of 40 °C and constant flow rate at 0.2 mL/min. The chromatographic gradient-based separation started with a solvent composition of 70% ultrapure water with 0.1% formic acid and 30% methanol for 3 min. After a further 3 min, the methanol content was steadily increased to 70%, and at 22 min, the methanol content was set to 95%. This composition was held for 3 min; afterward, within 1 min, the solvent ratio was set to the starting condition, which was maintained until the end of the 30 min run. The quantification was carried out using an external, SIL-based 9-point calibration series ranging from 0.001 to 3.0 mg/L per native analyte (for TB 0.0001 to 0.3 mg/L) at a concentration of 0.1 mg/L per SIL (for TB-D5, 0.01 mg/L) and calibration point. Blanks and control samples were measured regularly (at least after every 10th injection) for quality assurance. Data acquisition and data evaluation of LC–MS measurements were performed with Agilent MassHunter Workstation Software ‘LC/MS Data Acquisition’ (version B.06.01), ‘Qualitative Analysis’ (version B.07.00), and ‘Quantitative Analysis’ (version 11).

Film preservatives from facade extracts (acidic extraction) were quantified using an HPLC system equipped with a UV/VIS detector (Waters Corporation Milford, MA, USA) as described by Kiefer et al. (2024). Briefly, target compounds were separated on a C-18 column (ACQUITY UPLC BEH C18, 2.1 mm × 150 mm, 1.7 µm + guard column 2.1 mm × 5 mm, 1.7 µm, both Waters Corporation, Wexford, Ireland) using a solvent gradient at a constant flow rate of 0.2 mL/min and a column temperature of 40 °C. For separation, a gradient ranging from 70% ultra-pure water containing 0.1% formic acid and 30% methanol increased to 80% methanol was applied. The analytes were quantified using an external 12-point calibration series, in the range from 0.01 to 15 mg/L per analyte, at defined wavelengths which were selected based on full UV–VIS spectra acquisitions: 240 nm for TB, 280 nm for MIT, CMIT, OIT and DCOIT, 317 nm for BIT, and 227 nm for the internal standard Mecoprop. As further quality assurances, blanks and control samples were measured regularly (at least after every 10th injection). The instrument control and data evaluation were conducted using the Waters-software ‘Empower3’ (version 7.00.00.99).

### Ecotoxicity tests of facade eluates on aquatic model organisms

Ecotoxicological effects were evaluated using the model organisms green algae (*Desmodesmus subspicatus*) and luminescent bacteria (*Vibrio fischeri*). For this purpose, from each leaching test, two pools were created; one by combining the eluates from the early elution cycles 1 to 4 as well as one from the later cycles 5 to 9. These pooled samples were added to the model organisms at various dilution levels reaching from 1:1 to 1:96.

#### Growth inhibition test of facade eluates on luminescent bacteria

The ecotoxicological test of facade eluates on *V. fischeri* was carried out according to DIN EN ISO 11348–2 (European Committee for Standardization, 2009b) as described by Kiefer et al. (2024). Three different batches of *V. fischeri* were cultivated and independently treated as biological triplicates. In glass cuvettes, 0.2 mL of the bacterial suspension was mixed with 0.8 mL of the undiluted eluate pool sample or the control solutions. After 30 min incubation at 15 ± 1 °C, the luminescence, as an indicator of cell growth or growth inhibition, was measured using a luminometer (Lumistox, Dr. B. Lange, Berlin, Germany). A serial sample dilution was performed (maximum dilution 1:96) and the luminescence was measured after 15 min of incubation at 15 ± 1 °C. The first dilution level of a sample that caused less than 20% inhibition compared to the growth control was defined as the lowest ineffective dilution (LID) value. Potassium dichromate at 4.9 mg/L and 2% sodium chloride solutions were used as positive and negative controls, respectively.

#### Growth inhibition test of facade eluates on green algae

To assess the toxicological impact of the generated facade eluates on green algae (SAG Göttingen, Strain Number 86.6), a growth inhibition test was conducted according to DIN 38412 L33:1991–03 as described by Kiefer et al. (2024). A *D. subspicatus* culture was first cultivated and then exposed to a serial dilution of the generated eluate pool samples (maximum dilution 1:96) or control solutions. Finally, the chlorophyll fluorescence was measured as an indicator of algae growth or growth inhibition using a fluorometer (Fluorescence-Spectrophotometer, Hitachi, Modell F-2700). For this, 3 mL of the inoculum solution was mixed with 24 mL of the serially diluted eluate pool samples or control solution, along with 3 mL of nutrient solution. The fluorescence of three independent triplicates was measured after 72 h of incubation at 23 ± 1 °C and pH = 7. The LID value was determined as the lowest dilution level at which the fluorescence measured was less than 20% compared to the control group. Deionized water and 0.6 mg/L potassium dichromate were used as negative and positive controls, respectively.

### Statistics

Graphical representations (except for Fig. 1) and statistical analyses were carried out using ’OriginPro 2019’ (version 9.6.0.172). The Kologoriv-Smirnov test was applied to assess the normal distribution of the data within each replicate series. To identify statistically significant differences between treatments and specimen variations, pairwise *T*-tests of independent samples were performed at a 95% significance level (*α* = 0.05).

## Results

### Encapsulation increases the remaining concentrations in facades for OIT and DCOIT post-leaching and irradiation, whereas for TB, exclusively post-leaching

**Fig. 2:**
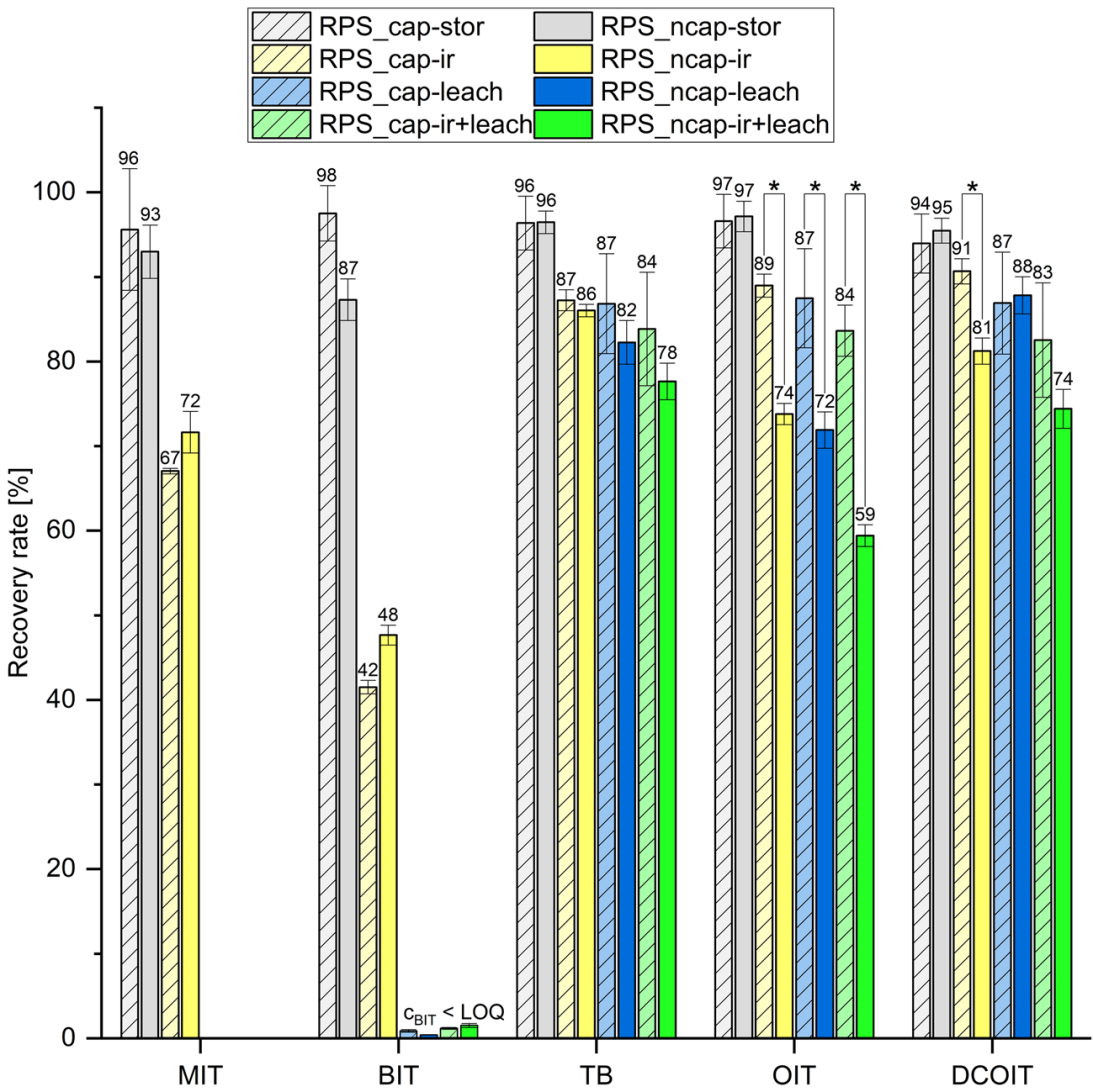
Relative recovery rates of non-encapsulated in-can preservatives (MIT, BIT) and either encapsulated or non-encapsulated film preservatives (TB, OIT, DCOIT) from the facade extracts of two different test facade variants (RPS_cap, hatched; RPS_ncap, blank). The recovery rates, calculated based on the experimentally analyzed initial concentrations, were determined after the following simulated environmental conditions: irradiation (ir, yellow, *n* = 1 × 3(analytical × technical replicate), leaching (leach, blue, *n* = 3 × 3), irradiation and leaching in combination (ir + leach, green, *n* = 3 × 3), storage as control (stor, gray, *n* = 1 × 3). Significant (*p* < 0.05) differences that were determined using a *T*-test for independent samples are marked with an asterisk (*). Absolute biocide concentrations are provided in Supplementary table S2

To investigate the effect of the encapsulation of film preservatives against UV irradiation and leaching, biocide-free control test facades (contr) and two different biocide-containing test facade variants were tested, containing either encapsulated (cap) or non-encapsulated (ncap) film preservatives. In contrast, in-can preservatives (BIT, MIT, and CMIT) were always added in non-encapsulated form. After 2 weeks with or without artificial sunlight irradiation in a weathering chamber, the UV-induced degradation and the leaching behavior were determined according to a standardized leaching test (DIN EN 16105). The relative recovery rates, obtained from the analyzed facade extracts as mass reference (mg/kg), were directly compared between the sample variants RPS_cap and RPS_ncap for each treatment (storage (stor), irradiation (ir), leaching (leach), irradiation + leaching in combination (ir + leach) (Fig. 2)). The original starting concentrations (c_0_) of each sample variant were determined at the beginning of the study, ensuring the comparability of the resulting relative concentrations after the different treatments. The biocide-free control samples were identically processed and analyzed, wherein, apart from low concentrations of BIT (maximum quantity of 23 mg/kg), no further biocides could be detected.

Comparing the different facade variants RPS_cap and RPS_ncap after irradiation, the always non-encapsulated compounds MIT and BIT showed comparable degradation levels leading to remaining concentrations of 72 ± 3% (RPS_ncap) and 67 ± 0% (RPS_cap) for MIT as well as 48 ± 2% (RPS_ncap) and 42 ± 2% (RPS_cap) for BIT. After an additional leaching test, no residual concentrations of MIT nor BIT could be quantified in the facade extracts. Furthermore, CMIT was not detectable in any test samples following any form of exposure.

Although TB was irradiated in both encapsulated and non-encapsulated states, comparable concentrations were measured in the facades after irradiation (RPS_cap, 87 ± 1%; RPS_ncap, 86 ± 1%). Also, no significant differences were determined between RPS_cap and RPS_ncap after the leaching test, irrespective of whether the test facades were irradiated before or not. However, both the mean recovery rates after leaching (RPS_cap 87 ± 6% vs. RPS_ncap 82 ± 3%) and leaching plus irradiation in combination (RPS_cap 84 ± 7% vs. RPS_ncap 78 ± 2%) appeared to be lower if TB had been non-encapsulated. Overall, the data on TB suggest that the encapsulation of this film preservative did not significantly impact its UV degradation. The leaching behavior seems to be slightly affected.

For OIT, the average recovery rates of the encapsulated substances were 15% higher after irradiation (RPS_cap, 89 ± 1%; RPS_ncap, 74 ± 1%) as well as leaching (RPS_cap, 87 ± 6%; RPS_ncap, 72 ± 2%) and even 25% higher after combined exposure (RPS_cap, 84 ± 3%; RPS_ncap, 59 ± 1%). Thus, in contrast to TB, encapsulation revealed an apparent protective effect for OIT against all types of simulated environmental exposure.

The same protective effects were observed for DCOIT after irradiation, with recovery rates of 91 ± 1% (RPS_cap) compared to 81 ± 2% (RPS_ncap). However, this effect was not as apparent when DCOIT was exposed to a combination of irradiation and leaching, where only a general tendency towards a protective impact of encapsulation is recognizable based on the mean recovery rates (RPS_cap, 83 ± 7%; RPS_ncap, 74 ± 3%). An effect of the encapsulation on the leaching behavior of DCOIT could not be observed (RPS_cap, 87 ± 6%; RPS_ncap, 88 ± 2%).

### Encapsulation and irradiation reduce leached concentration of the film preservatives TB and OIT

The effect of encapsulation on biocide release into eluates with and without irradiation was investigated with an immersion test. The concentrations measured after storage or irradiation, relative to the surface area (mg/m^2^), were considered as the initial concentrations for the leaching test. Of the three applied in-can preservatives, BIT, MIT, and CMIT, the most abundant compound was BIT with a relative concentration of 98.2 ± 0.4%. In contrast, CMIT could not be detected after storage or irradiation.

**Fig. 3:**
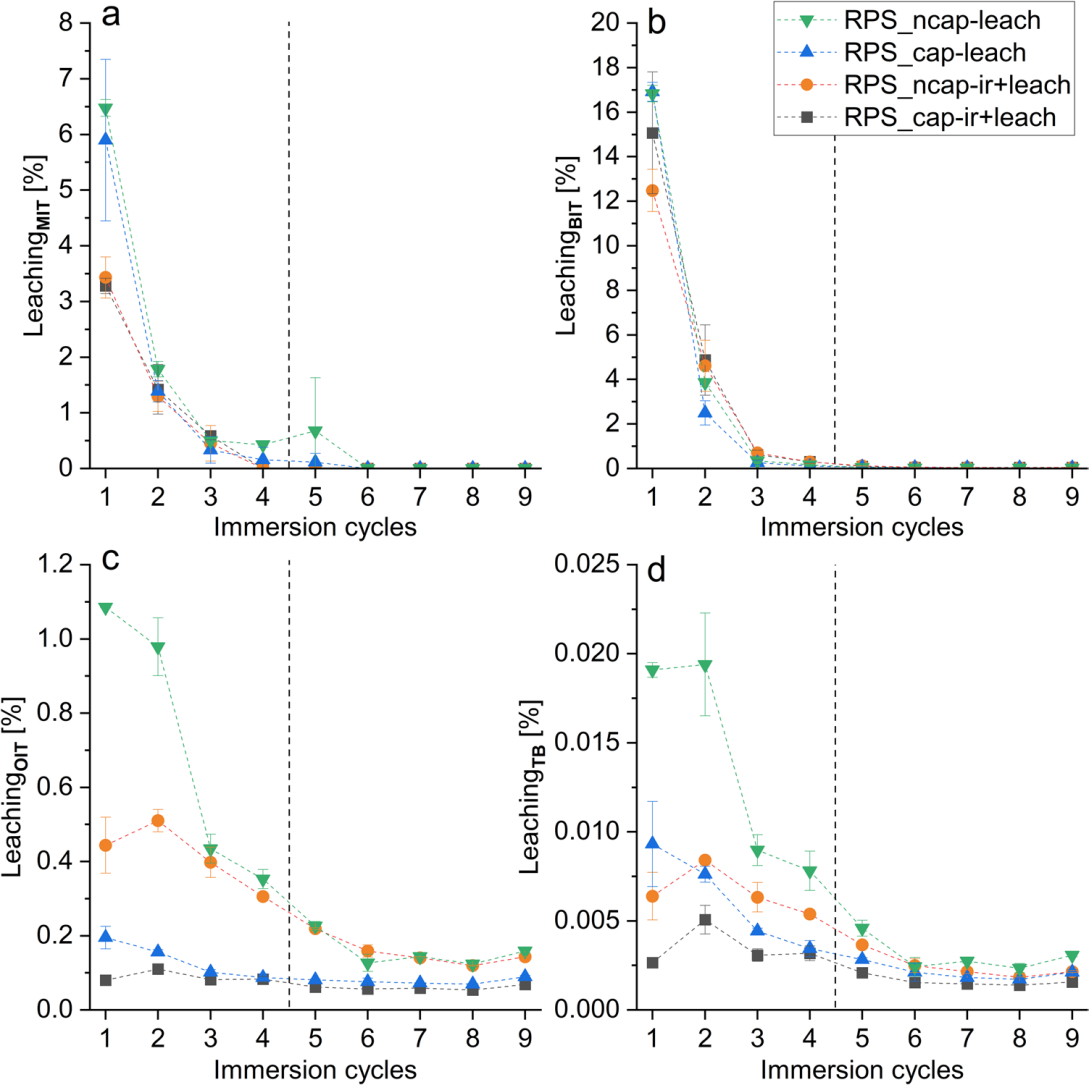
Leaching curves of the in-can preservatives MIT (**a**) and BIT (**b**) as well as the film preservatives OIT (**c**) and TB (**d**) according to the immersion test DIN EN 16105 given as percentage leaching amount per immersion cycle (1–9, n_A_ = 3 per cycle). The leaching procedure was performed on two different test sample variants, each with non-encapsulated in-can preservatives and either non-encapsulated (RPS_ncap) or encapsulated (RPS_cap) film preservatives. Before the immersion test, facade samples were either stored in the dark (leach) or artificially irradiated (ir + leach). The dashed line symbolizes the divided pool samples of cycles 1–4 and 5–9 which were used for toxicological tests

The non-encapsulated in-can preservatives tended to leach almost entirely during the first four immersion cycles, irrespective of previous treatments (Fig. 3a, b). For the non-irradiated test samples, 8 ± 2% (RPS_cap) and 10 ± 1% (RPS_ncap) of the initial MIT concentration were detected in the eluates, with the majority present in the eluate of the first immersion cycle (RPS_cap, 75%; RPS_ncap, 65%). After irradiation, the total amount of MIT leached decreased for both variants of facades to 5 ± 1%, with the largest proportion present in the eluate of the first cycle (RPS_cap, 62%; RPS_ncap, 66%). The leaching behavior of BIT was highly similar to that of MIT. However, the total leached-out BIT amount across all test sample variants and treatments was broadly constant between 18 ± 2% (RPS_ncap-ir) and 21 ± 5% (RPS_cap-ir). Again, first cycles showed the highest concentrations, peaking at 68% (RPS_cap) after irradiation and 85% (RPS_ncap) after storage. In this regard, irradiation reduced BIT leaching by 14% (stor, 85%; ir, 71%) and 11% (stor, 79%; ir, 68%) for the test sample variants RPS_cap and RPS_ncap, respectively.

In contrast, the film preservatives in all samples leached out in low but sustained concentrations throughout the nine immersion cycles (Fig. 3c, d). Furthermore, TB and OIT showed a slightly delayed leaching behavior after irradiation, regardless of the test facade variant. The highest quantities were observed in the eluates of the second immersion cycle for both OIT (RPS_ncap, 21%; RPS_cap, 17%) and TB (RPS_ncap, 22%; RPS_cap, 23%). The leaching trends for TB and OIT are mainly comparable, demonstrating reduced leaching concentrations caused by the influence of irradiation and encapsulation. The highest cumulative leaching rate over all cycles was observed for the non-encapsulated and non-irradiated OIT, amounting to 3.6 ± 0.2% of the initial concentration. Irradiation decreased the total leached amount of non-encapsulated OIT to 2.4 ± 0.2%. However, encapsulation had an even greater effect on the leaching behavior, lowering the eluted quantity to 0.9 ± 0.1%. Additionally, irradiation considerably reduced the total leaching rate to 0.7 ± 0.1% for encapsulated OIT. Analogous to the findings for OIT, the greatest extent of leaching over the entire test period was observed for non-encapsulated and non-irradiated TB, amounting to 0.070 ± 0.007% of the initial concentration. Both irradiation of the non-encapsulated substance and encapsulation without irradiation reduced the total amount of TB leached out by about half to 0.040% (± 0.003–0.004%). Encapsulated and irradiated TB showed the lowest leaching behavior, with 0.020% (± 0.003%) of the starting concentration. The film preservative DCOIT used in the test samples could not be detected in any eluate sample.

### Encapsulation reduces ecotoxicity of facade eluates on luminescent bacteria and green algae without and with irradiation

**Fig. 4:**
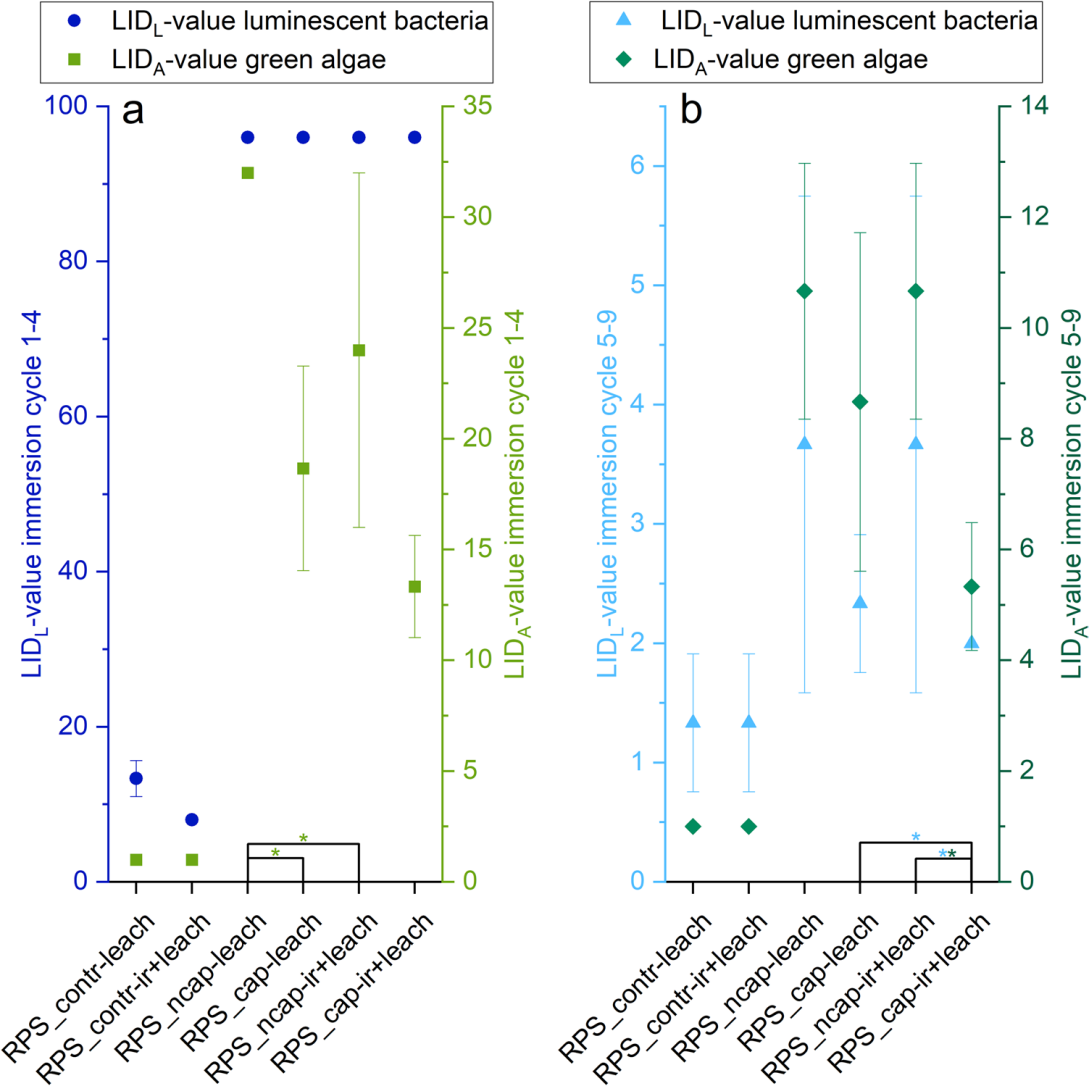
Toxicity of faced eluates for green algae (A, olive green (**a**) and dark green (**b**)) and luminescent bacteria (L, dark blue (**a**) and light blue (**b**)) given as lowest ineffective dilution (LID) value. Presented are the values for the pooled eluates of the immersion cycles 1–4 (**a**) and 5–9 (**b**). Eluates were generated according to the immersion test DIN EN 16105 using different test facade variants which contained non-encapsulated in-can preservatives and non-encapsulated (RPS_ncap) or encapsulated film preservatives (RPS_cap) or by biozide-free control facades (RPS_contr). Before the leaching test, facade samples were weather irradiated (ir + leach) or stored in the dark (leach). Significant differences determined using a T-test for independent samples are marked with an asterisk (*). LID values are provided in Supplementary table S3

To evaluate the impact of irradiation and encapsulation on the ecotoxicity of facade eluates for the initial and later stages of the leaching process, the eluates generated in the immersion test were collected for each test sample variant (RPS_contr, RPS_cap, RPS_ncap), treatment (leach, ir + leach), and replicate (*n*_A_ = 3). The eluates from immersion cycles 1–4 as well as 5–9 were combined, chemically characterized, and tested for toxicity on green algae and luminescent bacteria (Fig. 4, details provided in Supplementary table S3).

For both pooled samples, the biocides BIT, MIT, CMIT, TB, OIT, and DCOIT were tested (Table 1), of which the biocides CMIT and DCOIT could not be detected. Throughout the first four immersion test cycles, the biocides were released in higher quantities, particularly the in-can preservatives MIT and BIT. For these two biocides, 99 to 100% of their total eluted amounts were washed out within the first half of the immersion test, regardless of the sample variant or treatment. The average eluted amounts for the pooled cycles 1–4 was 75 ± 5% for TB and 69 ± 10% for OIT. The pooled eluates from the immersion test cycles 5–9 generally contained lower biocide concentrations and were dominated by the film preservatives. In contrast, MIT could not be detected in the pooled eluates of cycles 5–9 and the leached-out BIT concentration was between 0.5 and 1%. In the eluates of cycles 1–4, small amounts of BIT could be determined in the biocide-free control samples; furthermore, TB and OIT could be detected below the quantification limit.

**Table 1:** Average leached-out biocide concentrations and standard derivation (*n*_A_ = 3) during the immersion test for each sample variant (RPS_contr, biocide-free; RPS_cap, encapsulated film preservatives; RPS_ncap, non-encapsulated film preservatives) and treatment (leach, leaching; ir, irradiation) of the pooled eluates of the cycles 1–4 and 5–9. *n.d* not detectable

	Immersion cycle 1–4 [mg/L]							
	MIT	± SD	BIT	± SD	TB	± SD	OIT	± SD
RPS_contr-leach	n.d		0.15	0.009	n.d		< LOQ	
RPS_contr-ir + leach	n.d		0.06	0.005	n.d		< LOQ	
RPS_ncap-leach	0.021	0.003	4.2	0.53	0.006	0.001	0.36	0.05
RPS_cap-leach	0.021	0.002	3.7	0.23	0.0019	0.00007	0.055	0.0006
RPS_ncap-ir + leach	0.012	0.002	2.1	0.15	0.0028	0.0002	0.17	0.007
RPS_cap-ir + leach	0.011	0.002	1.7	0.36	0.0010	0.0001	0.04	0.002
	Immersion cycle 5–9 [mg/L]							
	MIT	± SD	BIT	± SD	TB	± SD	OIT	± SD
RPS_contr-leach	n.d		n.d		n.d		n.d	
RPS_contr-ir + leach	n.d		n.d		n.d		n.d	
RPS_ncap-leach	n.d		0.022	0.001	0.0014	0.00006	0.084	0.004
RPS_cap-leach	n.d		0.018	0.003	0.0006	0.000007	0.033	0.001
RPS_ncap-ir + leach	n.d		0.020	0.001	0.0010	0.00003	0.063	0.0005
RPS_cap-ir + leach	n.d		0.022	0.002	0.0004	0.00003	0.028	0.001

Despite the independent production and slightly different initial concentrations of RPS_cap and RPS_ncap facade samples, general trends in biocide composition and the effects of encapsulation and irradiation are evident from the total amounts of biocide leached. The eluate samples from the first half of the leaching test were quantitatively dominated by BIT, with concentrations ranging from 1.7 mg/L (RPS_cap-ir + leach) to 4.2 mg/L (RPS_ncap-leach). In contrast, the quantified values for MIT ranged from 0.011 mg/L (RPS_cap-ir + leach) to 0.021 mg/L (RPS_(n)cap-leach), for OIT from 0.04 mg/L (RPS_cap-ir + leach) to 0.36 mg/L (RPS_ncap-leach), and for TB from 0.001 mg/L (RPS_cap-ir + leach) to 0.006 mg/L (RPS_ncap-leach). In the second half of the leaching test, in-can preservatives were insignificant, with BIT detected at only 0.02 mg/L and MIT undetectable in all samples. The concentrations of the leached film preservatives also decreased for the pooled eluates from the leaching cycles 5–9, with emitted quantities ranging from 0.028 mg/L (RPS_cap-ir + leach) to 0.084 mg/L (RPS_ncap-leach) for OIT and 0.0004 mg/L (RPS_cap-ir + leach) to 0.0014 mg/L (RPS_ncap-leach) for TB. As a general trend, it can be concluded that the absolute amount of biocide leached is reduced due to encapsulation and irradiation. Considering the biocide-free control samples, low levels of BIT and unquantifiable amounts of OIT were found in the eluates of leaching cycles 1–4. CMIT and DCOIT were undetectable in any sample despite being present in the starting formulation of the biocide-containing test facades.

All immersion samples from the biocide-containing test facades (RPS_cap and RPS_ncap) in the first half of the leaching test (cycles 1–4, Fig. 4a) exhibited the maximum toxicity value (lowest ineffective dilution (LID)) of 96, regardless of sample variant or treatment. This indicates that bacterial toxicity was mainly driven by in-can preservatives, primarily BIT, which comprised 99% of the analyzed preservatives. For the samples of the second half of the immersion test (cycles 5–9, Fig. 4b), the toxicity value was below 4 in all cases. The highest LID values were observed for the eluates of the non-encapsulated facade sample variants after and without irradiation (for both 3.7 ± 2.1), while the lowest toxicity was found for the encapsulated samples (leach, 2.3 ± 0.6; ir + leach, 2.0 ± 0). Furthermore, the eluates from the irradiated RPS_cap test facades were significantly less toxic than those from the non-irradiated RPS_cap sample and the irradiated RPS_ncap variant. Additionally, the control samples showed slightly increased toxicity values for the pooled eluates from cycles 1–4 with LID values of 8 ± 0 and 13 ± 2.3 for the RPS_contr-ir + leach and RPS_contr-leach, respectively. For both samples, the value dropped to 1 ± 0 for the pooled eluates from cycles 5–9 (Fig. 4).

Generally, the algae toxicity assay resulted in lower LID values for the eluates from the first half of the immersion test (cycles 1–4, Fig. 4a) compared to the luminescent bacteria assay. In contrast, the eluates from the second half of the test (cycles 5–9, Fig. 4b) showed higher algae toxicity levels, excluding the control samples. As an overall trend, the eluates from the test facades containing non-encapsulated film preservatives exhibited higher algae toxicity levels, with LID values of 32 ± 0 and 24 ± 8 without and after irradiation, respectively, for the first half of the immersion test. The algae toxicity value decreased to 10.7 ± 2.3 for both eluate samples (RPS_ncap-leach, RPS_ncap-ir + leach) of the second half of the immersion test. Without irradiation, encapsulation of the film preservatives reduced the toxicity level of the eluates from the first (cycles 1–4) and second (cycles 5–9) half of the immersion test to 18.7 ± 4.6 and 8.7 ± 3.1, respectively. Additionally, irradiation of the test sample variants RPS_cap-ir + leach decreased the toxicity to 13.3 ± 2.3 and 5.3 ± 1.2 for cycles 1–4 and 5–9, respectively. Both halves of the immersion test showed that eluates from RPS_ncap-leach samples had significantly higher toxicity than those from RPS_ncap-ir + leach and RPS_cap-leach variants. The irradiated and encapsulated samples were significantly less toxic than the non-irradiated and non-encapsulated variants. Control samples had a LID value of 1 ± 0 for each eluate.

## Discussion

### Leaching of film preservatives TB, OIT, and DCOIT is reduced by encapsulation

The leaching behavior of non-encapsulated biocides has already been extensively studied and has been summarized comprehensively in tabular form by Paijens et al. (2020). In contrast, encapsulated film preservatives were only systematically studied through leaching by Vermeirssen et al. (2018), focusing on altered elution but not considering irradiation, which was consequently addressed in our study.

After performing the leaching test, neither MIT nor CMIT and only non-quantifiable amounts of BIT could be determined within the tested facade matrix. The rapid leaching of the in-can preservatives from facade test samples has already been observed in various analogous studies and can primarily be attributed to the relatively high water solubility of the substances (Kiefer et al. 2024; Schoknecht et al. 2009). The leaching behavior of the in-can preservatives resulted in high biocide loads in the facade eluates, if only for a limited time. Nevertheless, the determined accumulated amounts of in-can preservatives within the facade eluates were lower than expected, regardless of the test sample variant and the treatment, as only 5–10% of the MIT starting concentration and around 20% of the BIT starting concentration were found in the eluates. Previous studies in which the same leaching procedure was carried out on comparable dispersion-based test facades documented a total discharge of BIT within the leachates ranging from 27 to 48% (Schoknecht et al. 2009) and up to 59% (Kiefer et al. 2024), respectively. The high mass differences can be explained in part by degradation processes taking place within the facade matrix and in the eluates. Isothiazolinones are generally considered to be volatile, thermally unstable and susceptible to extreme pH conditions (Silva et al. 2020; Edge et al. 2001). The study by Rafoth et al. (2007) demonstrated that BIT dissolved in tap water degraded by approximately 90% and almost linear within 14 days at room temperature and under UV protection. During the same period, CMIT degraded almost completely, and MIT could no longer be quantified after the seventh day. Additionally, there are indications that an alkaline environment, as it is present within the facade matrix and for the facade eluates, could promote the degradation of MIT, CMIT, and BIT. Barman and Preston (1992) investigated the influence of the pH value on the hydrolytic degradation of MIT and CMIT, finding the lowest half-life of the two substances to be two to three days at a basic pH value between 9.6 and 10. Krzeminski et al. (1975) concluded from their results that the hydrolytic degradation of CMIT is accelerated with increasing pH and temperature values, with a half-life of about six days at a pH of 9.0 and 40 °C. Wang et al. (2017) irradiated dissolved BIT at a wavelength of 254 nm under different pH values and observed a faster BIT degradation at a pH of 10, with a kinetic constant of 0.17 min^−1^, compared to a higher kinetic constant of 0.03 min^−1^ and a slower BIT degradation at a pH of 5.

According to the recovery rates determined within the test facades after the leaching test, the encapsulation tends to provide a protective effect for TB and showed an apparent significant protective effect for OIT, whereas no difference between the encapsulated and non-encapsulated variants could be determined for DCOIT. The findings suggest that the effectiveness of encapsulation against the leaching from facade samples is enhanced with an increased water solubility of a substance. However, it cannot be excluded that the active substances were encapsulated with different technologies or polymers, resulting in different protective effects. Based on the leaching curves prepared following the DIN EN 16105 test, a clear protective effect of the encapsulation for TB and OIT was observed. The total amount of TB and OIT emitted fell from 0.07 to 0.04% and from 3.6 to 0.9% respectively as a result of the encapsulation. DCOIT could probably not be detected in any eluate due to the low washout rates below the detection limit (0.05 mg/L). The protective effect of encapsulation for film preservatives against leaching is consistent with previous studies (Nordstierna et al. 2010; Vermeirssen et al. 2018; Jämsä et al. 2013). Vermeirssen et al. (2018) carried out the same leaching procedure in a slightly modified form (5 L/m^2^ instead of 25 ± 5 L/m^2^ eluate volume and constant shaking during the test), finding that encapsulation reduced the total leached amounts of the film preservatives TB, OIT, and DCOIT from 3.4 to 0.9% (TB), from 12 to 0.7% (OIT), and from 0.3 to 0.01% (DCOIT), respectively. In the studies by Kiefer et al. (2024), the total emitted discharge of encapsulated TB and OIT after the DIN EN 16105 leaching test was 5.3% and 17.2%, respectively. Quantitative differences in the total leached-out amounts across different studies can likely be attributed to variations in the chemical and physical properties of the test facades, since factors such as the affinity between active substance and matrix, amount of dissolved organic carbon, porosity, and surface structure can influence leaching behavior (Schoknecht et al. 2009).

### Encapsulation significantly increases the stability of OIT and DCOIT in facades

The UV-induced degradation of biocides has been the focus of several scientific studies so far; however, these investigations often take place under artificial and/or simplified laboratory conditions (aqueous standard solutions, UVC radiation, accelerated treatments). Especially the degradation pathways and mechanisms of biocides in building materials and particularly in facade matrices remain largely unclear and mass balances are often incomplete (Urbanczyk et al. 2019; Bollmann et al. 2016, 2017b). In this context, in-can preservatives play a comparatively minor role compared to film preservatives, as in-can preservatives have a shorter life span and are less frequently detected in the environment (Kiefer et al. 2024). A more complex but realistic alternative to laboratory tests is the outdoor weathering of test facades. Although field trials provide findings that can be transferred to real-world scenarios, it is challenging to draw definitive conclusions about the underlying causes and effects due to the multifactorial environmental influences. For example, it is impossible to differentiate between hydrolytic and photolytic degradation during an outdoor test.

The exposure of the facade samples to artificial solar radiation led to considerably extensive degradation of BIT (58% and 52%) compared to MIT (only 33% and 28%) in facades with and without encapsulated film preservatives, most probably due to the higher UV-absorption rate of the emitted UV-radiation (295–400 nm) of BIT with two absorption maxima at 225 nm and 320 nm compared to the absorption maximum of MIT at 275 nm. Overall, the similar recovery rates of MIT or BIT between the two test sample variants (encapsulated and non-encapsulated) suggest a comparable UV degradation, indicating no notable influence from the encapsulation materials of the film preservatives. It can be assumed that the photoinduced UV degradation primarily takes place in the upper coating layers of the facade system, so that lower biocide amounts are discharged within the first leaching cycle after irradiation. Due to the recurring water contact with intermediate drying phases during the leaching test procedure, the resulting concentration gradients caused by prior irradiation were equalized over time by diffusion-driven processes (Schoknecht et al. 2009, 2013). Consequently, the photodegradation in the upper coating layer, the consequent reduction in leached amounts during the first leaching cycle, and the overall low initial and therefor limited MIT concentrations collectively suggest that the photodegradation/irradiation may have had a relevant influence on the leaching behavior of MIT.

After irradiation of the facade samples, significantly higher recovery rates were found for the encapsulated variants of the film preservatives OIT and DCOIT, whereas no differences were observed between encapsulated and non-encapsulated TB. This suggests that encapsulation primarily offers a protective effect against UV radiation for isothiazolinone-based preservatives. OIT and DCOIT have their UV-absorption maximum at 275 nm each, whereas TB shows its absorption maximum at 225 nm and therefore does not or just slightly absorb the emitted UV radiation from 295 to 400 nm (Urbanczyk et al. 2019; Junginger et al. 2022). This is further consistent with the results for the non-encapsulated biocides, where TB exhibited the highest recovery rates within the facade matrix after UV exposure. Urbanczyk et al. (2019) assumed that TB degradation in dispersion-based paint matrices is not a direct result of UV radiation, but rather due to indirect photolysis reactions initiated by excited polymers. Additionally, the researchers proposed that the polymers, which make up a significant component within dispersion-based paints and renders, form polymer radicals through photolytic reactions and thus contribute directly and indirectly, e.g., through the formation of reactive oxygen species, to biocide degradation.

For the worst-case scenario involving irradiation and leaching of the test facades, again a significant protective effect of encapsulation was determined for OIT, whereas for TB and DCOIT, protection provided by encapsulation can only be assumed based on higher averaged recovery rates. This is probably because encapsulation did not affect the UV degradation of TB and had no impact on the leaching behavior of DCOIT within the test facades. Overall, the encapsulation of the film preservatives resulted in different protection efficiencies depending on the active ingredient and the treatment, which is probably due to the different chemo-physical properties of the analytes, such as water solubility and absorption behavior, as well as various applied encapsulation processes and encapsulation polymers.

Based on the progression of the leaching curves, clear influences of irradiation were observed for both the encapsulated and non-encapsulated film preservatives. Regardless of the encapsulation, the relative maximum amounts leached out for TB and OIT were not detected in the eluates of the first leaching cycle as for the in-can preservatives, but in the eluates of the subsequent leaching cycle. This suggests UV degradation of the biocides in the upper coating layer of the test facades, leading to lower leached out amounts during the first leaching cycle, followed by concentration equalization through subsequent diffusion processes within the facade matrix due to the wet and dry phases of the leaching test. Overall, the relative maximum and total released amounts during the leaching test decrease after irradiation of the test facades. This observation applies to both the encapsulated and non-encapsulated variants of TB and OIT. The results show that the lowest amounts of biocide are emitted from a facade system when the active ingredient is encapsulated in combination with prior radiation, which is close to real-world conditions.

### Encapsulation of TB, OIT, and DCOIT decreases the toxicity of facade eluates on luminescent bacteria and green algae

The extensive leaching of the in-can preservatives led to high biocide levels in the pooled facade eluates from the leaching cycles 1–4, primarily dominated by BIT. Consequently, the maximum inhibition values obtained on luminescent bacteria across all samples can likely be attributed to the high BIT concentrations within the tested eluates. BIT is known to possess both fungicidal and bactericidal properties (Varga et al. 2020; Silva et al. 2020). Despite a decrease of about 50% in the BIT concentration within the eluate samples of cycles 1–4 after irradiation of the test facades, no reduction in toxicity to the luminescent bacteria was observed, demonstrating the potent inhibitory effect of the biocide and the sensitivity of the luminescent bacteria test to BIT. The assumption that BIT is the primary driver causing the growth inhibition of the luminescent bacteria is supported by the results of the pooled eluates from the leaching cycles 5–9. The toxicity levels of the eluates generated from the biocide-containing facade samples, now containing substantially reduced but comparable BIT concentrations, were only slightly higher compared to the eluates of the biocide-free control samples. Nevertheless, the different toxicological effects of the pooled eluates from leaching cycles 5–9 on the luminescent bacteria are correlated with the emitted OIT concentrations. Encapsulation reduced the leached OIT concentration without and after irradiation of the test facades by around 60%, while the toxicological value fell from 4 to 2. Although irradiation of the non-encapsulated facade variant resulted in a decreased OIT discharge in the second half of the leaching cycle, this did not lead to a change in the toxicological effect. The amounts of TB washed out were not considered further for the evaluation of the luminescent bacteria test, as there is no indication of a significant inhibitory effect of TB on luminescent bacteria (Vermeirssen et al. 2018; Bollmann et al. 2017a). The elevated toxicological impacts observed for the control samples generated from the biocide-free declared test facades on the luminescent bacteria assay can be attributed to the low BIT concentrations and non-quantifiable levels of OIT present within the eluates of the immersion cycles 1–4. Kiefer et al. (2024) also conducted toxicological testing of dispersion-based facade eluates on luminescent bacteria and found that the presence or absence of film preservatives did not impact the growth of the luminescent bacteria. They concluded that the in-can preservative BIT was the primary driver of the toxicological effect. Further studies examining the impact of single or combined film preservatives without the presence of in-can preservatives (TB or OIT (Bollmann et al. 2017a); TB, OIT, and DCOIT (Vermeirssen et al. 2018)) on luminescent bacteria identified OIT as the primary growth inhibitor.

Based on the known algaecidal properties of TB (Kresmann et al. 2018; Reiß et al. 2021), this substance is assumed to be the primary factor responsible for the algal toxicity observed in the eluate samples. Across the entire toxicological evaluation, a clear trend emerged, where eluates with higher absolute TB concentrations exhibited greater inhibitory effects on green algae growth. Particularly, the results of the eluate samples from leaching cycles 1–4 demonstrate that encapsulation and irradiation of the facade samples reduced the absolute amount of leached-out TB, resulting in decreased growth inhibition of the green algae. Furthermore, the green algae test results reveal a similar pattern for the eluates of immersion cycles 5–9 as the results for the luminescent bacteria. While irradiation of the non-encapsulated facade variant slightly decreased the discharged TB concentration, this did not lead to a notable change in the toxicological impact. Consistent with the findings from the first half of the leaching test, the encapsulation of TB and irradiation of the encapsulated test sample variant resulted in the lowest total TB amount within the eluates and, consequently, the weakest inhibitory effect on algal growth. Also, Vermeirssen et al. (2018) observed that encapsulated TB exhibited reduced leaching compared to the non-encapsulated variant, resulting in reduced toxicity to green algae. Additionally, they excluded the isothiazolic film preservatives OIT and DCOIT as potential inhibitors of photosystem II and, consequently, as drivers of the toxicological effect on green algae. In summary, eluates with elevated TB concentrations caused greater inhibitory growth effects on the green algae. The highest absolute amounts of TB were detected in the eluate sample generated from the non-irradiated and non-encapsulated facade variant. Furthermore, the results demonstrate that encapsulation and irradiation of the test facades reduced the total TB emitted, thereby decreasing the toxicological impact.

## Conclusion

In summary, it was demonstrated that encapsulation of the film preservatives TB and OIT notably diminishes their leaching from facades. Furthermore, encapsulation significantly enhances the stability of OIT and DCOIT in facades under UV irradiation. After the combined treatment of irradiation and leaching, encapsulation proved to be significantly protective for OIT within the facade matrix. A comparable trend and an apparent protective impact by encapsulation were also observed for TB and DCOIT. As a consequence, it was also shown that encapsulation considerably lowers the toxicity of facade eluates in particular of the later, not by BIT dominated eluates on luminescent bacteria and green algae. These finding holds true for both the non-irradiated and the even fewer toxic eluates of the irradiated facades. Consequently, the encapsulation of film preservatives in facade materials extends their sustainability and lessens the ecotoxicological implications for water organisms caused by facade eluates.

## Supplementary Information

Below is the link to the electronic supplementary material. 

ESM1 (DOCX 1.35 MB)

## Data Availability

The authors state that the data supporting the study’s findings are included in the paper and in the supplementary information files. If raw data in another format or presentation is required, it can be obtained from the corresponding author upon request.
