# Supplementary material for: Encapsulated biocides in facade materials impact leaching and UV stability, resulting in lower aquatic toxicity of the eluates
Source: Environ Sci Pollut Res Int. 2025 Jun 25;32(27):16324–39. doi: 10.1007/s11356-025-36647-2 (PMC12274237; doi:10.1007/s11356-025-36647-2)
Supplement: Supplementary file 1 — (DOCX 1.35 MB) [file 11356_2025_36647_MOESM1_ESM.docx]

**Supplementary Information (SI): Encapsulated biocides in facade materials impact leaching and UV stability, resulting in lower aquatic toxicity of the eluates (Journal: Environmental Science and Pollution Research)**

**Moritz Nichterlein^1,2,*^** (ORCID: 0000-0002-6992-6691), **Nadine Kiefer^1,2,*^** (ORCID: 0000-0002-6716-8188) **, Jenny Hohner^1^, Dominik Stapf^1^, Madeleine Schatz^3,4^, Matthias Noll^1^** (ORCID: 0000-0002-9981-5968)**, Stefan Kalkhof^1,2,5^** (ORCID: 0000-0001-6121-7105)

^1^Institute for Bioanalysis, University of Applied Sciences Coburg, Germany

^2^Institute for Analytical Chemistry Universität Leipzig, Leipzig, Germany ^3^SGS Analytics Germany GmbH, Augsburg, Germany
^4^Limbach Analytics GmbH, Leipzig, Germany
^5^Proteomics Unit, Fraunhofer Institute for Cell Therapy and Immunology, Leipzig, Germany

* These authors contributed equally.

Correspondence:

Stefan Kalkhof
Institute for Bioanalysis, Department of Applied Sciences,

Coburg University of Applied Sciences and Arts, 96450 Coburg,

E-mail address: [Stefan.kalkhof@hs-coburg.de](mailto:Stefan.kalkhof@hs-coburg.de)

**Table S1** Composition (w/w %) of the dispersion-based render and paint formulations used for the production of the facade test samples (Render-Paint-Systems; RPS_contr, RPS_ncap, RPS_cap). The render and paint formulations of the biocide-containing test sample variants (RPS_ncap and RPS_cap) were additionally treated with biocide formulations (ACTICIDE from Thor, Speyer, Germany)

| Ingredients | Description | Render | Paint |
| --- | --- | --- | --- |
|  |  | Composition as mass weight [%] | |
| Water |  | 9.8 | 18.98 |
| Tylose H 10000 P2 | Hydroxyethylcellulose | 0.1 |  |
| Tylose MH 30000 YG8 | Methylhydroxyethylcellulose |  | 0.19 |
| Bentone EW | Rheological additive, Magnesium Lithium Silicate | 0.2 |  |
| Dispex AA 4135 | Polymeric dispersing agent based on acrylic acid sodium salt | 0.3 | 0.28 |
| Sodium hydroxide 20% | Alkaline solution | 0.2 | 0.19 |
| Byk 012 | Defoamer on polymer-basis (VOC-free and silicone-free) | 0.2 | 0.28 |
| Acronal ECO 6716 im IBC | Aqueous dispersion of a styrene acrylate polymer | 7.0 |  |
| Vinnapas 822 HD | Polymer-dispersion of a terpolymer of vinyl chloride, ethylene and vinyl ester in water | 7.0 |  |
| Titandioxid Kronos 2160 | Rutile pigment, Titanium dioxide treated with Al and Si | 2.0 | 9.30 |
| Omyacarb 40 GU | Calcium carbonate | 18.0 |  |
| Omyacarb 130 GU | Calcium carbonate | 16.0 |  |
| Omyapearl 500/10-SV | Calcium carbonate | 10.0 |  |
| Arbocel B 400 | Natural cellulose fiber | 0.2 |  |
| Carolith 1,5 - 2,0 mm | Calcium carbonate | 29.0 |  |
| Glimmer MU-75 R | Rheological additive, Silicate mixture |  | 4.50 |
| Omyacarb 5 GU | Calcium carbonate |  | 14.00 |
| Omyacarb 15 GU | Calcium carbonate |  | 23.00 |
| Acronal ECO 6716 | Aqueous dispersion of a styrene acrylate polymer |  | 28.00 |
| Silres BS 1306 | Silicone oil emulsions; Water-dilutable emulsion of a polysiloxane modified with functional silicone resin |  | 1.00 |
| Coapur 5535 | Solvent-free non-ionic liquid polyurethane thickener |  | 0.28 |


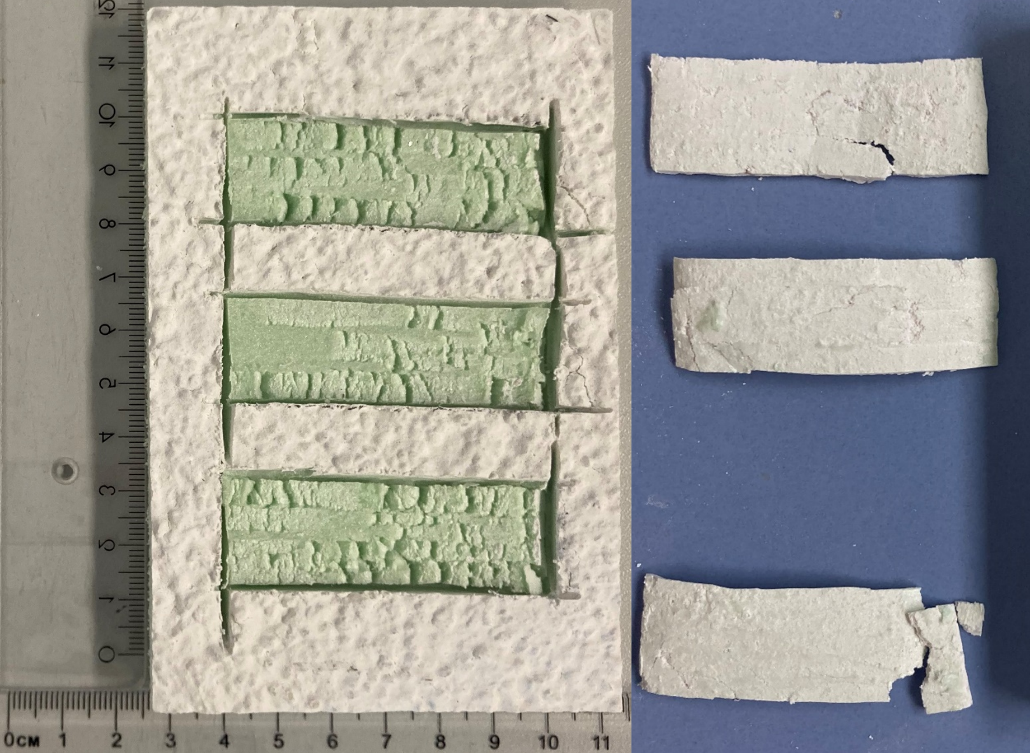


**Figure S1** Facade test samples (Render Paint System: RPS; insulation layer of polystyrene (green), plaster (white), paint (white)) after sampling to determine the biocide concentrations within the facade matrix. The samples (n_T_ = 3) taken from one facade test sample were pooled and processed as technical replicates.

|  |  |  | RPS_contr | | | | | | RPS_ncap | | | | | | RPS_cap | | | | | |
| --- | --- | --- | --- | --- | --- | --- | --- | --- | --- | --- | --- | --- | --- | --- | --- | --- | --- | --- | --- | --- |
|  |  |  | MIT | CMIT | BIT | TB | OIT | DCOIT | MIT | CMIT | BIT | TB | OIT | DCOIT | MIT | CMIT | BIT | TB | OIT | DCOIT |
| MIT, CMIT, BIT: Methanoilc extraktion, LC-MS TB, OIT, DCOIT: Acidic extraction, HPLC-UV/VIS | c_0_ (n_A_=1; n_T_=3): | MW [mg/kg] | 1 | n.d. | 27 | n.d. | n.d. | n.d. | 8 | <LOQ | 673 | 374 | 430 | 383 | 9 | n.d. | 657 | 287 | 442 | 377 |
|  | Starting concentration | MW [mg/m2] | 5 | n.d. | 164 | n.d. | n.d. | n.d. | 48 | <LOQ | 3867 | 2151 | 2474 | 2202 | 47 | n.d. | 3307 | 1444 | 2226 | 1899 |
|  |  | SD [%] | 1 |  | 4 |  |  |  | 4 |  | 1 | 2 | 1 | 2 | 1 |  | 4 | 3 | 3 | 1 |
|  | -stor (n_A_=1; n_T_=3): | MW [mg/kg] | 1 | n.d. | 23 | n.d. | n.d. | n.d. | 8 | n.d. | 587 | 361 | 418 | 366 | 9 | n.d. | 640 | 276 | 427 | 354 |
|  | After storage (no irradiation) | MW [mg/m2] | 5 | n.d. | 137 | n.d. | n.d. | n.d. | 45 | n.d. | 3376 | 2075 | 2403 | 2102 | 45 | n.d. | 3225 | 1397 | 2158 | 1790 |
|  |  | SD [%] | 1 |  | 4 |  |  |  | 3 |  | 2 | 1 | 2 | 2 | 6 |  | 3 | 2 | 2 | 2 |
|  | -ir (n_A_=1; n_T_=3): | MW [mg/kg] | 1 | n.d. | 15 | n.d. | n.d. | n.d. | 6 | n.d. | 321 | 322 | 318 | 311 | 6 | n.d. | 273 | 250 | 393 | 342 |
|  | After irradiation without leaching | MW [mg/m2] | 4 | n.d. | 93 | n.d. | n.d. | n.d. | 35 | n.d. | 1843 | 1850 | 1826 | 1789 | 31 | n.d. | 1373 | 1260 | 1980 | 1722 |
|  |  | SD [%] | 1 |  | 7 |  |  |  | 3 |  | 2 | 1 | 2 | 2 | 0.4 |  | 2 | 1 | 2 | 2 |
|  | -leach (n_A_=3; n_T_=3): | MW [mg/kg] | n.d. | n.d. | n.d. | n.d. | n.d. | n.d. | n.d. | n.d. | <LOQ | 308 | 309 | 336 | n.d. | n.d. | <LOQ | 249 | 387 | 328 |
|  | After leaching without irradiation | MW [mg/m2] | n.d. | n.d. | n.d. | n.d. | n.d. | n.d. | n.d. | n.d. | <LOQ | 1695 | 1704 | 1853 | n.d. | n.d. | <LOQ | 1211 | 1862 | 1567 |
|  |  | SD [%] |  |  |  |  |  |  |  |  |  | 3 | 3 | 2 |  |  |  | 7 | 7 | 7 |
|  | -ir+leach (n_A_=3; n_T_=3): | MW [mg/kg] | n.d. | n.d. | n.d. | n.d. | n.d. | n.d. | n.d. | n.d. | <LOQ | 291 | 256 | 285 | n.d. | n.d. | <LOQ | 240 | 370 | 311 |
|  | After irradiation and leaching | MW [mg/m2] | n.d. | n.d. | n.d. | n.d. | n.d. | n.d. | n.d. | n.d. | <LOQ | 1670 | 1470 | 1638 | n.d. | n.d. | <LOQ | 1254 | 1947 | 1650 |
|  |  | SD [%] |  |  |  |  |  |  |  |  |  | 3 | 2 | 3 |  |  |  | 8 | 8 | 8 |

**Table S2** Absolute biocide concentrations (mg/kg and mg/m^2^) of the facade test samples (Render-Paint-System; RPS_contr, RPS_ncap, RPS_cap) before (c_0_: starting concentration) and after the treatments carried out (stor: storage in the dark, ir: irradiation, leach: leaching, ir+leach: irradiation and leaching). The biocide-containing facade sample variants (RPS_ncap, RPS_cap) each contained non-encapsulated in-can preservatives (MIT, CMIT, BIT; measurement with LC-MS) as well as non-encapsulated (RPS_ncap) or encapsulated (RPS_cap) film preservatives (TB, OIT, DCOIT; measurement with HPLC-UV/VIS). n.d.: not detectable, n_A_: analytical replicates, n_T_: technical replicates

**Table S3** Toxicity of faced eluates given as lowest ineffective dilution (LID) value for luminescent bacteria (L) and green algae (A). Eluates were generated according to the immersion test DIN EN 16105 using different test facade variants which contained non-encapsulated in-can preservatives and non-encapsulated (RPS_ncap) or encapsulated film preservatives (RPS_cap) or by biozide-free control facades (RPS_contr). Before the leaching test, facade samples were weather irradiated (ir+leach) or stored in the dark (leach). Samples from cycles 1-4 and 5-9 were pooled and measured as analytical replicates (n_A_ = 3).

|  | Pooled eluates of the immersion cycles 1-4 | | | | Pooled eluates of the immersion cycles 5-9 | | | |
| --- | --- | --- | --- | --- | --- | --- | --- | --- |
|  | LID_L_-value | ± SD | LID_A_-value | ± SD | LID_L_-value | ± SD | LID_A_-value | ± SD |
| RPS_contr-leach | 13.3 | 2.3 | 1.0 | 0.0 | 1.3 | 0.6 | 1.0 | 0.0 |
| RPS_contr-ir+leach | 8.0 | 0.0 | 1.0 | 0.0 | 1.3 | 0.6 | 1.0 | 0.0 |
| RPS_ncap-leach | 96.0 | 0.0 | 32.0 | 0.0 | 3.7 | 2.1 | 10.7 | 2.3 |
| RPS_cap-leach | 96.0 | 0.0 | 18.7 | 4.6 | 2.3 | 0.6 | 8.7 | 3.1 |
| RPS_ncap-ir+leach | 96.0 | 0.0 | 24.0 | 8.0 | 3.7 | 2.1 | 10.7 | 2.3 |
| RPS_cap-ir+leach | 96.0 | 0.0 | 13.3 | 2.3 | 2.0 | 0.0 | 5.3 | 1.2 |
